# Interpretation of convolutional neural networks reveals crucial sequence features involving in transcription during fiber development

**DOI:** 10.1186/s12859-022-04619-9

**Published:** 2022-03-15

**Authors:** Shang Liu, Hailiang Cheng, Javaria Ashraf, Youping Zhang, Qiaolian Wang, Limin Lv, Man He, Guoli Song, Dongyun Zuo

**Affiliations:** 1 Institute of Cotton Research of Chinese Academy of Agricultural Sciences, Anyang, 455000 China; 2 Zhengzhou Research Base, State Key Laboratory of Cotton Biology, Zhengzhou University, Zhengzhou, 450001 China; 3 Department of Plant Breeding and Genetics, University College of Agriculture and Environmental Sciences, The Islamia University of Bahawalpur, Punjab, 63100 Pakistan

**Keywords:** Cotton fiber, Transcription, Convolutional neural network, Model interpretation, Motif detection

## Abstract

**Background:**

Upland cotton provides the most natural fiber in the world. During fiber development, the quality and yield of fiber were influenced by gene transcription. Revealing sequence features related to transcription has a profound impact on cotton molecular breeding. We applied convolutional neural networks to predict gene expression status based on the sequences of gene transcription start regions. After that, a gradient-based interpretation and an N-adjusted kernel transformation were implemented to extract sequence features contributing to transcription.

**Results:**

Our models had approximate 80% accuracies, and the area under the receiver operating characteristic curve reached over 0.85. Gradient-based interpretation revealed 5' untranslated region contributed to gene transcription. Furthermore, 6 DOF binding motifs and 4 transcription activator binding motifs were obtained by N-adjusted kernel-motif transformation from models in three developmental stages. Apart from 10 general motifs, 3 DOF5.1 genes were also detected. In silico analysis about these motifs’ binding proteins implied their potential functions in fiber formation. Besides, we also found some novel motifs in plants as important sequence features for transcription.

**Conclusions:**

In conclusion, the N-adjusted kernel transformation method could interpret convolutional neural networks and reveal important sequence features related to transcription during fiber development. Potential functions of motifs interpreted from convolutional neural networks could be validated by further wet-lab experiments and applied in cotton molecular breeding.

**Supplementary Information:**

The online version contains supplementary material available at 10.1186/s12859-022-04619-9.

## Background

Upland cotton (*Gossypium.hirustum.*L) takes up about 90% of cotton cultivated over the world and is the main crop contributing to renewable textile fibers [1]. Fiber development of upland cotton could be divided into four stages: initiation, elongation, secondary cell wall thickening (SCW), and maturity. Agronomic traits of fiber are mainly formed in the first three stages, and corresponding genes related to fiber formation are also transcripted in these stages [2–4]. Genome assembly of upland cotton enables researchers to perform high throughput transcriptome analysis and fetch gene sequences efficiently [2–5]. Given that gene transcription is the base of phenotype formation and the genome sequences are the base of heredity, it’s of significant meanings to disclose transcription-related sequence features for molecular breeding. In maize, convolutional neural networks (CNNs) were applied to the prediction of relative transcriptional abundance and roles of untranslated regions (UTR) in transcription were revealed [6]. The application of CNN in maize inspired us to utilize CNNs in upland cotton to detected sequence features related to transcription.

CNNs have been applied to predict binding sites of transcription factors or RNA-binding proteins [7–11]. For the binding sites prediction tasks, convolutional neural networks (CNN) showed good performance in accuracy. DeepBind used chromatin immunoprecipitation sequencing (CHIP-seq) and crosslinking-immunoprecipitation sequencing (CLIP) to predict binding sites of transcription factors and RNA binding proteins, respectively [11]. DeepSEA utilized CHIP-seq datasets, DNase I–hypersensitive sites, and histone-mark profiles to identify binding sites of transcription factors and accessibility of chromatin [10]. In these models, input sequence was one-hot encoded as a 1-D sequence with 4 channels (A, T, C, G). The encoded sequences were dealt with models to get a binding score which presented the binding ability of the transcription factor. Successful applications of these models in protein-sequence binding prediction indicate that CNNs are suitable for dealing with genome sequences. Apart from the high accuracy reached by CNNs, the other advantage CNNs possess is the ability for motif detection, which could interpret models’ parameters into sequence features with biological meanings [8].

Motif detection implemented by interpretation of CNNs has been tried in several types of research about a prediction of protein-sequence binding chromatin accessibility [8, 10–12]. In these previous studies, filters in the first convolutional layer were supposed as motif scanners and selected for interpretation. Strategies for kernel transformation in these studies are similar. Searching for activated regions of sequences is implemented. Subsequently, activated regions selected by several criteria were pooled together. Finally, sequences within activated regions were used to calculate a position weight matrix (PWM) through the cross-entropy method. PWMs are aligned to the database in JASPAR for known motifs detection, while unaligned PWMs will be detected as novel motifs [12]. In these model interpretation strategies, PWMs were generated from activated regions within sequences. Compared with other interpretation methods, such as DeepLIFT, SHAP, and saliency analysis which calculate significant scores for single nucleotide, model interpretation performed by generating PWMs could provide sequence features in the form of biological meanings [13–15]. Protein-DNA (RNA) binding prediction is required for recognizing binding sites from context sequences and evaluating the effects of variants within binding sites, so generating PWMs from sequences is a reasonable interpretation strategy. But in the field of predicting transcription status, we aim to detect motifs influencing transcription and have no demand for evaluation of variants within sequences, so we could apply another kernel-transformed strategy for interpretation of CNN models.

Input sequences of CNNs are one-hot encoded, which means parameters of kernels in the first convolutional layer represent for effects of channels (A, T, C, G) in activated regions, and the values of kernel parameters could be normalized into a PWM. However, in a trained model, final parameters are influenced by the initialization of model and the process of model training. To adjust bias result from parameter initialization and model training in kernel transformation, we proposed an N-adjusted kernel-transform method. Generally, apart from 4 types of nucleotides (A, T, C, G), N also exists within genomic sequences and these N characters are masked repeat elements or cryptic nucleotides in the genome [16]. In our opinion, these N characters provide little information for prediction task, and if parameters of N channel are not equal to 0, we regard the value possessed by N as bias generated from model initialization and training.

We constructed CNNs with a single convolutional layer for it is competent for motif detection [12]. 10 motifs that potentially involve in fiber development were detected by N-adjusted kernel transformation and several novel sequence features were also interpreted from trained models. Adjusted N kernel-transformation, a strategy to interpret kernels directly into PWMs by N adjusted, could be applied in other prediction tasks implemented by CNNs. Sequence features detected in this study could be applied as sequence markers for gene selection in cotton molecular breeding. All the source code in this study is available at https://github.com/LiuShang-777/SingleCNN.

## Results

### Samples were classified into three clusters

To gain training data for CNN models, we implemented transcriptome sequencing on ovules and fiber from 11 time points. The reliability of our transcriptome data was validated by Pearson coefficient between three biological replicates and 33 temporal samples showed good reproducibility of transcriptome atlas (See Additional file 1). Interestingly, we found that these samples could be divided into three clusters according to the heatmap. To classify gene expression patterns during fiber development, we applied the t-distributed stochastic neighbor embedding (t-SNE) method on transcriptome data. The mean FPKM value of three biological replicates was calculated for t-SNE analysis and result of t-SNE could be viewed in an additional dot plot (See Additional file 2). Results of t-SNE suggested initiation, elongation, and SCW were covered in the transcriptome atlas. Samples from different developmental stages had various transcriptional patterns, which indicated models should be built separately for three stages.

### CNN predicted gene expression status accurately

**Fig. 1:**
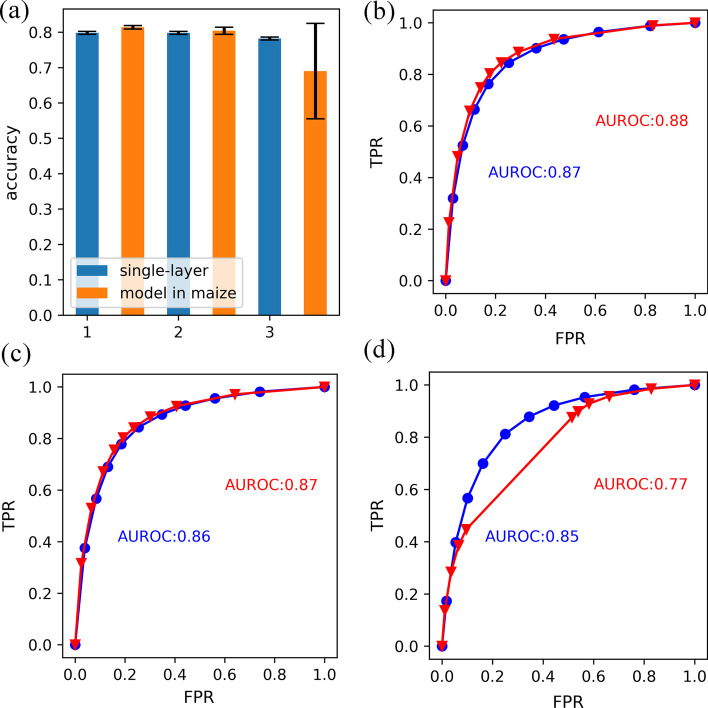
Accuracies and ROC curves of models with single convolutional layer and models in maize for three stages. **a** Accuracies of models in three stages, blue bars represent for accuracies of our models and orange bars represent for accuracies of models in maize. **b** ROC curve for our model and the model in maize for fiber initiation, X-axis is FPR (false positive rate) and Y-axis is TPR (true positive rate). Red line for model in maize and blue line for our model. **c** ROC curve for models in fiber elongation. **d** ROC curve for models in SCW

Three kinds of CNN models with different parameters were built corresponding to initiation, elongation, and SCW. Sequences and expression data were preprocessed according to Method. To gain models of high quality, we had applied 210 combined parameters (7 for number of filters, 6 for kernel size and 5 for max-pooling size). Average accuracy on fivefold cross validation was used to evaluate performance of parameters and we selected models with different parameters for 3 stages (Table 1 and Additional files 3, 4, 5). To compare with models applied in maize, we constructed the previously reported model and trained it with our transcriptome data [6]. Accuracies and areas under the receiver operating characteristic curve (AUROC) were used to evaluate model performance. As shown in Fig. 1a, average accuracies of our models in initiation and elongation reached about 80% having no significant divergence with models applied in maize (81% and 80% in initiation and elongation, respectively). However, models reported in maize had an extremely low accuracy (about 52%) in SCW and the average accuracy of models was 70%. Our models in SCW had a higher average accuracy (about 78%) and no extremely low accuracy. Apart from average accuracy, AUROCs of models in 3 stages were also compared (Fig. 1b, c). 2 types of models had no significant divergence on AUROC in initiation and elongation. Our models had higher AUROC in SCW. Average accuracy and AUROC suggested models with a single convolution layer are still competent for the prediction of gene expression status. The high quality models we gained enabled us to perform model interpretation for important sequence features.

**Table 1:** Selected models for three stages

Developmental stage	Number of filters	Kernel size	Maxpooling size	Average accuracy
Initiation	24	14	10	0.8
Elongation	32	22	10	0.8
SCW	24	16	10	0.78

Parameters and mean accuracy of corresponding models in three developmental stages. These three selected models had highest mean accuracy compared with other combined parameters in each stage

### 5′UTR is important for transcription

**Fig. 2:**
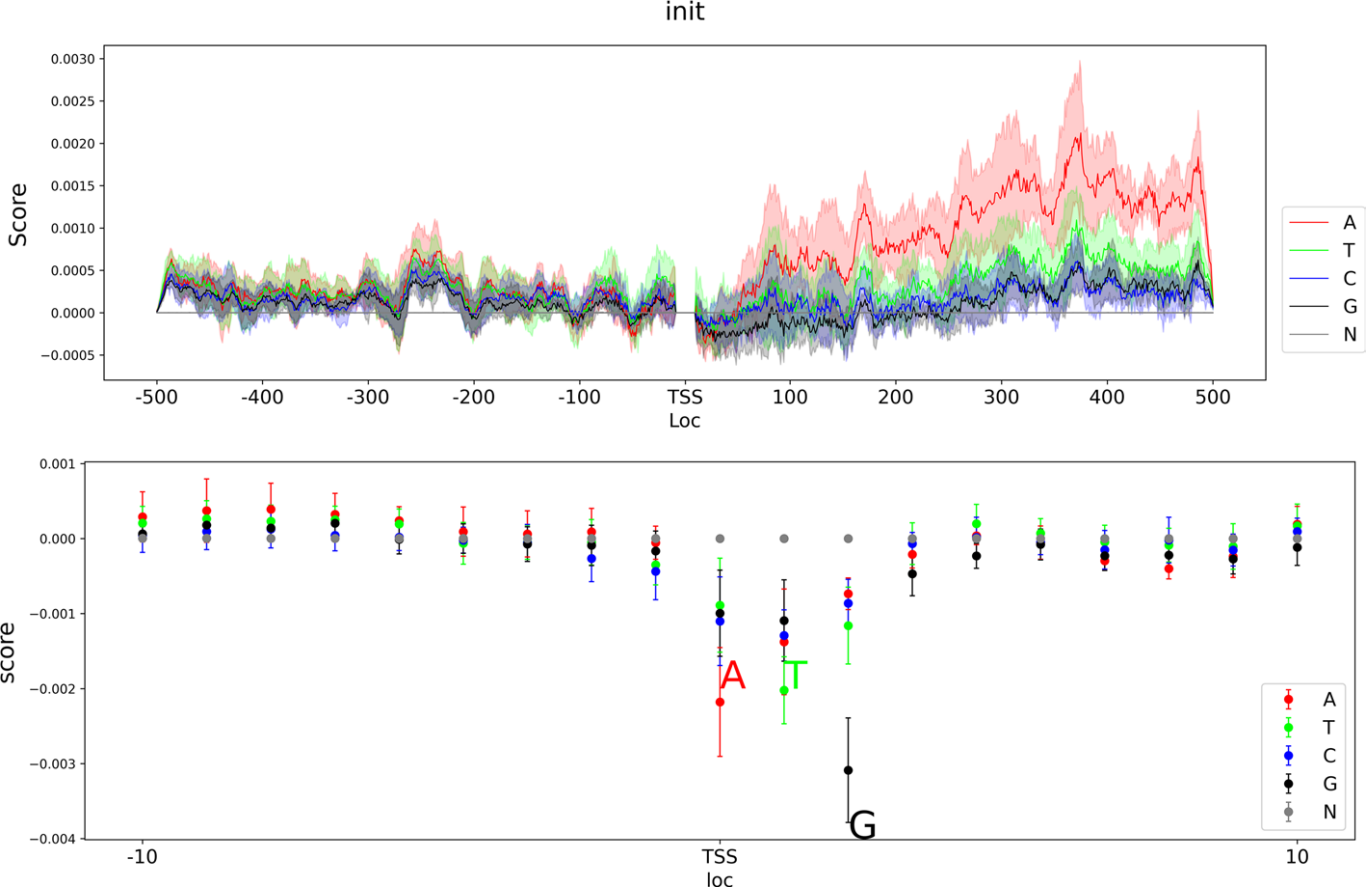
Average effect of each loc in input sequences. Plot visualized effects of 991 input features except for upstream and downstream 10nts from transcription start site. Scatter under the effects plot visualized effects of upstream and downstream 10 nts

Aiming to interpret models with biological meanings, we applied a published package named DeepLIFT which was previously used in biology field to interpret the models [6, 15, 17, 18]. We selected all true positive sequences (genes were correctly predicted as expressed) in three stages for interpretation. Figure 2 and Additional files 6 and 7 exhibited the average effect of each loc on the input sequence for models of initiation. To display extreme effects on TSS, we masked upstream and downstream 10 nucleotides (nts) from TSS and visualized them in a seperate dot plot.

We found that A, T, and G on the TSS had negative effects on transcription. Interestingly, a gene starting with trinucleotides ATG means it has no annotation for 5′UTR in the reference genome and these genes were always predicted by de novo prediction tools. Negative effects of ATG reflected that de novo predicted genes tended to have low transcription abundance or even be pseudogenes [19]. According to these results, we advised that in genome annotation, de novo prediction method should be substituted by methods based on multi-samples transcriptome data. Apart from effects on TSS, we also found downstream sequences (from TSS) had higher effects than those of upstream sequence. Downstream 500 nts contained UTR region of genes. Therefore, we inferred that 5′UTR may play an important role in gene transcription. Notably, within 5′UTR, A and T had relatively higher effects than C and G, demonstrating nucleotide A and T had potential contributions to gene transcription. We had found important sequence region and compared contribution of 4 types of nucleotides for transcription, however, we could not interpret specific motifs from results of DeepLIFT. To find out motifs of biological meanings, a motif transformation method should be applied in model interpretation.

### Kernel transformation detected transcription-related motifs effectively

**Fig. 3:**
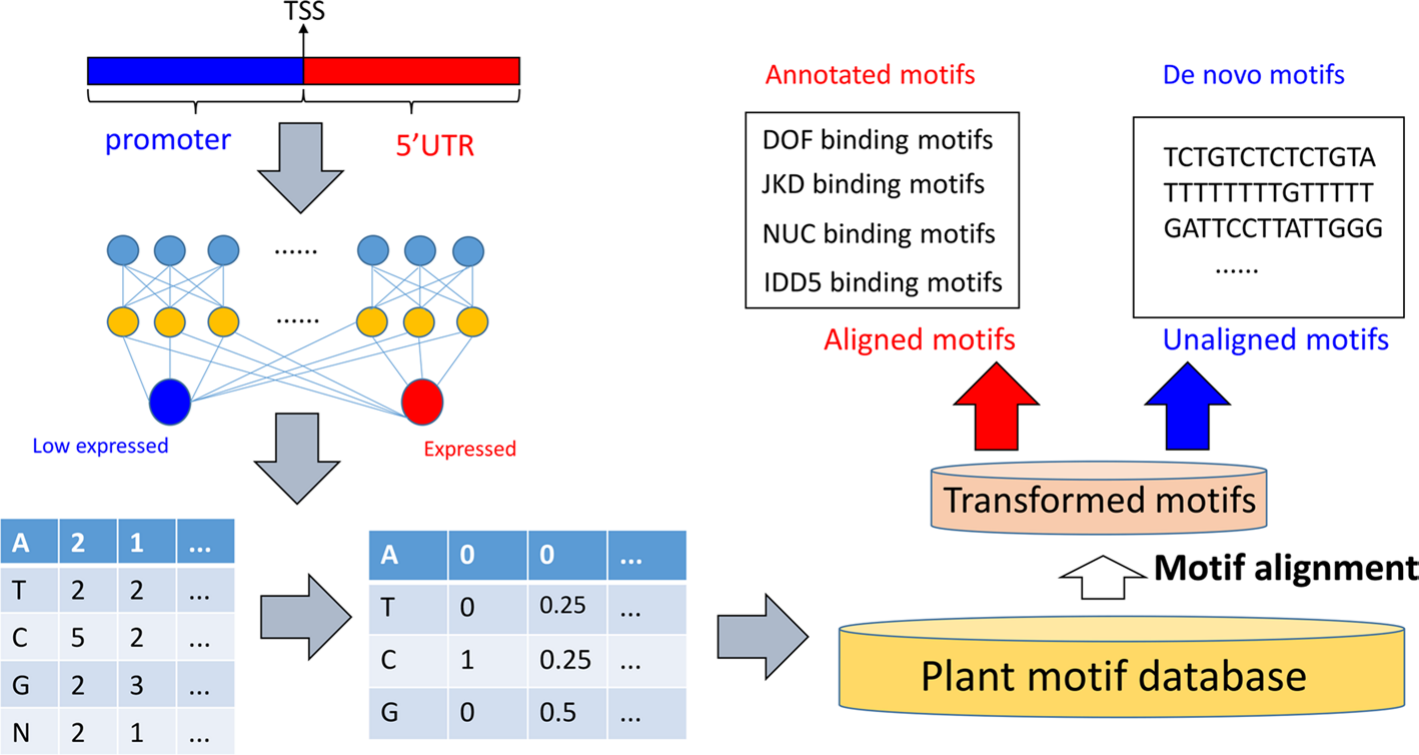
Pipeline to interpretate CNN models by adjusted kernel transformation. Kernels from CNN models will be transformed into PWM with N-regularized method. Transformed kernel will be aligned with known plant motifs to detect both annotated and de novo sequence features

**Fig. 4:**
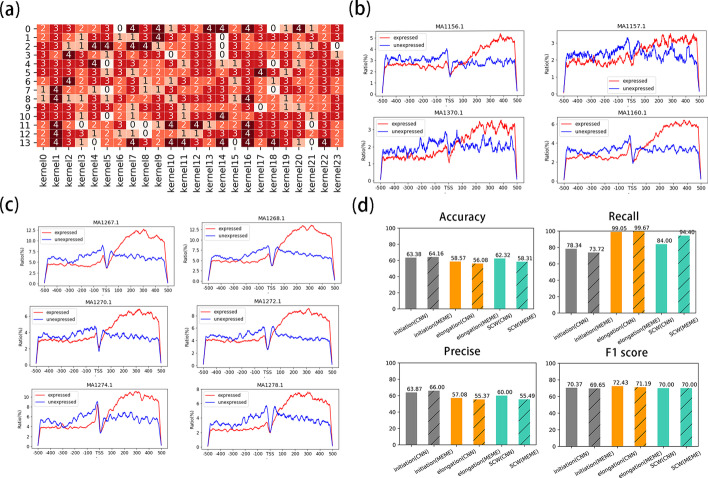
Model interpretation results by N-adjusted kernel transformation. **a** Visualization for effects of N in model0 during initiation. Values annotated in the heatmap indicate the order of N sorted by effects of 5 characters in sequences. 4 means N has the largest effect and 0 means effects of N is minimum among 5 characters. **b** Distribution for 4 transcription activators detected from kernel transformation on expressed (red line) and low expressed genes (blue line). **c** Distribution for 6 DOF genes detected from kernel transformation on expressed (red line) and low expressed genes (blue line). **d** Comparison among features extracted by CNN interpretation and meme. Features extracted by two methods were input into SVM model to predict transcription status. SVM models were evaluated in accuracy, recall rate, precise rate and f1 score. Bars with shadows represent for SVM build on features extracted by MEME and bars without features represent for SVM build on features detected from CNN

Kernels of CNN were regarded as motif scanners, these kernels could be transformed into motifs in the form of PWM [8]. We developed an adjusted kernel transformation method to transform kernels from CNN models into motifs. The description of this pipeline was displayed in Fig. 3. Patterns of all kernels in models were visualized in a set of additional files (See Additional files 8, 9, 10, 11, 12, 13, 14, 15, 16, 17, 18, 19, 20, 21, 22). All the kernels were transformed into motifs in the form of PWM by N-adjusted kernel transformation described in Methods. We introduce N as cryptic nucleotides in reference to be an effect control for other 4 nucleotides. Visualization of N’s effect order in all kernels showed that in several positions, the control effects were higher than those of normal nucleotides and these N present in reference genome should not be ignored in data preprocessing (Fig. 4a and Additional files 23, 24, 25). Our adjusted kernel transformation could adjust effects of 4 nucleotides by introducing N as a control to generate motifs. These transformed motifs were aligned to the non-redundant plants motif database in JASPAR [20]. For each developmental stage, if aligned motifs from the motif database present in at least 2 cross-validation models, they would be considered as common motifs, while, the others were assigned as specific motifs. We obtained 12, 36 and 22 common motifs in initiation, elongation and SCW, respectively. 10 common motifs were shared by 3 developmental stages and their distribution within sequences showed diverse patterns in expressed and low expressed genes implying these motifs had crucial roles for transcription (Additional file 26, Fig. 4b, c). Details about aligned motifs were recorded in Additional file 27.

To compare validity of motifs interpreted by adjusted kernel transformation method and traditional motif enrichment method, we collected enriched motifs on expressed genes with the MEME, and the most significant 12, 36, and 22 motifs (corresponding to number of the common motifs) in initiation, elongation, and SCW, respectively (See Additional file 27) [21]. We re-predicted gene expression through support vector machine (SVM) based on motifs extracted by kernel transformation and motifs enriched by MEME. SVM models were evaluated in 4 aspects, accuracy, recall rate, precise rate, and f1 score. Performance of two kinds of SVM was compared and visualized in Fig. 4d. The results showed motifs extracted by our kernel transformed method had similar effects as motifs obtained by the MEME illustrating that common motifs we gained had good reliability.

### In silico analysis implied crucial role of detected motifs in transcription

Among 10 shared common motifs, 6 of them could be bound by DOF transcription factors, while the other 4 motifs could be bound by 4 transcription activators, including 2 IDD5, NUC and JKD. 6 DOF genes were annotated as important factors involved in transcription. We built homologous models for 6 DOF factors in Arabidopsis and aligned them to the templates in SwissModel (See Additional file 28) [22]. According to alignment, second structures of 3 DOF factors (AT1G69570, AT2G28810, and OBP3) were similar to polymerase II subunit RPB9 implying these DOF factors may have a similar function as subunits of polymerase II in transcription [23, 24]. In Arabidopsis, JKD is an activation factor regulating asymmetric cell division, epidermal-cell-type patterning and stem cell maintenance [25, 26]. Functions of JKD in Arabidopsis are about cell division, elongation and cell wall formation indicating it may involve in initiation, elongation and SCW during fiber development, respectively. NUC was also reported as transcription activator related to asymmetric cell division in Arabidopsis [25].

**Fig. 5:**
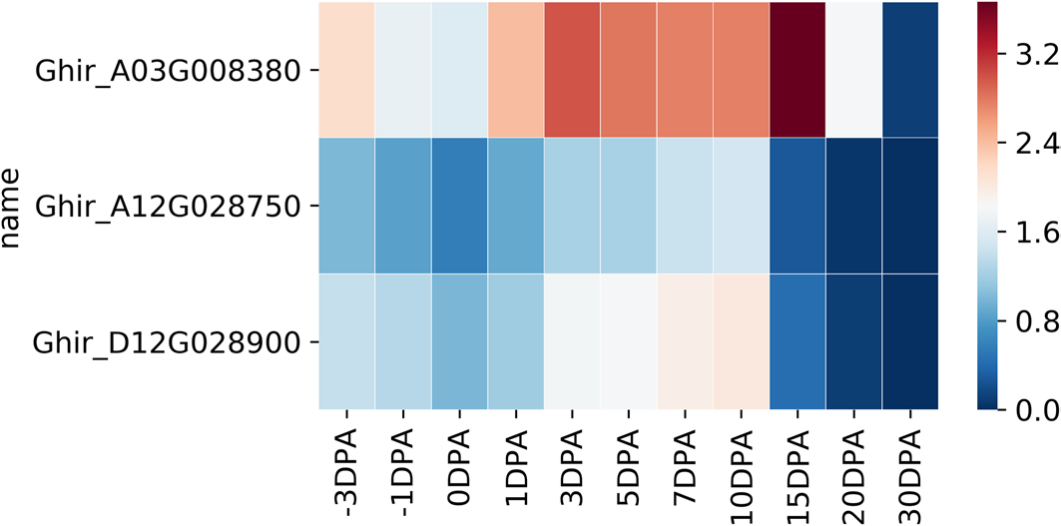
Heatmap of transcriptional abundance of DOF5.1 in upland cotton. Mean FPKM on each timepoint was calculated based on 3 replicates and log2 (FPKM + 1) was used for heatmap visualization

In common motifs which were not shared in three stages, we focused on DOF5.1 (AT5G02460) from elongation because it regulated auxin transport in Arabidopsis which could affect fiber elongation [27]. We visualized homologous genes of DOF5.1 in upland cotton and found that they had relatively higher transcriptional abundance in the stage of fiber elongation (Fig. 5). We inferred that DOF5.1 in upland cotton could influence fiber elongation through regulating auxin transportation.

### Novel motifs contribute to divergence between monocotyledon and dicotyledon

Apart from kernel-transformed motifs which were aligned to the motif database, there were still transformed motifs that could not aligned to the database and these motifs were assigned as de novo plant motifs. In each developmental stage, unaligned motifs were aligned to each other and a non-redundant de novo motif set was obtained after filtering aligned motifs. We scanned the upland cotton genome with non-redundant motifs to investigate distribution of these de novo motifs within genomic sequences. We found some of these de novo sequence features had different distribution patterns between two categories (expressed and low expressed genes) and we selected three of them from motifs detected during three developmental stages (Additional files 29, 30, 31). GO analysis of genes possessing these motifs indicated they were mainly involved in splicing, transportation, and localization of nucleic acids (Additional files 32, 33, 34). The essential biological preprocesses were enriched in genes with de novo motifs and this indicated de novo motifs we detect may play important roles in development of plants.

## Discussion

In this study, we applied CNNs to detect sequence features crucial for transcription during fiber development. Subsequently, we interpreted the models and extracted related sequence features involving transcription. Our CNNs had only one convolutional layer due to avaibility for interpretation and reduction of model complexities [12]. Compared with models in maize, accuracies and AUROCs in three stages illustrated CNNs with single convolutional layer was competent for task of transcription status prediction [6].

Here, we took a direct kernel-transformation strategy different from those applied in protein-DNA (RNA) binding prediction [7–12]. The core of predicting binding sites is to recognize a limited number of binding sites from context sequences. Given that, it is important to ensure kernels had no or lower activated values in context regions. What’s more, to evaluate about variants within binding sites, generating PWMs from sequences containing variants within activated regions is necessary. However, in prediction of expression status, nucleotides possessing significant positive scores take a high ratio on input sequence according to results of DeepLift and it’s inappropriate to generate PWMs from larger activated regions. As first convolutional layer of CNN was regarded as motif scanner, we normalized parameters of kernels into PWMs by N-adjusted kernel transformation. The visualization of N in kernels showed that nucleotides which should contribute little information for prediction could have higher effects compared with A, T, C, and G. Therefore, it will introduce bias to ignore character N in sequences and effects of 4 normal types of nucleotides in kernel transformation should be adjusted by effects of N. Functions of motif-binding proteins we gained by N-adjusted kernel transformation are about asymmetric cell division, auxin transport and transcription activation. Meanwhile, genes possessing novel motifs were enriched in GO terms about transportation, localization and splicing of nucleic acids which are essential for transcription. Both results indicate that we have detected potential motifs involving in transcription during fiber development.

In this study, we also found 5′UTR play important role in gene transcription which is corresponding to previous study and the role of 5′UTR in plant [28]. However, we found A and T had more contributions for transcription, while C and G were more important in maize. These divergent results may be caused by the limited sample size used in maize (high expressed gene and pseudogenes). During transcription, the unwinding of the DNA double helix is an essential step in which hydrogen bonds between nucleotides pair would be broken. A-T pairs possessing two hydrogen bonds are easier to be broken than C-G pairs having three hydrogen bonds. Therefore, genes with AT enriched 5′UTR need less energy to start transcription. We inferred high AT ratio within 5′UTR is a sequence feature of expressed genes.

## Conclusion

In summary, CNN with a single convolutional layer has reliable performance on the prediction of gene expression status. N-adjusted kernel transformation could extract important sequence features underlying sequences. Motif-binding proteins could be functionally validated by further wet-lab experiments and N-adjusted kernel transformation could be applied broadly in other crops to figure out crucial sequence features prompting molecular breeding.

## Methods

### Transcriptome sequencing and profiling

#### Plant materials preparation

The tetraploid upland cotton Texas Marker 1 (TM-1) was grown in the field belong to Institute of Cotton Research of Chinese Academy of Agricultural Sciences, located in Anyang, Henan province. Ovules were collected on 3 days before anthesis (-3 DPA), 1 day before anthesis (-1 DPA), anthesis (0 DPA), 1 day post-anthesis (1 DPA), 3 days post-anthesis (3 DPA), and 5 days post-anthesis (5 DPA). Fibers were collected on 7 days post-anthesis (7 DPA), 10 days post-anthesis (10 DPA), 15 days of anthesis (15 DPA), 20 days post-anthesis (20 DPA), and 30 days post-anthesis (30 DPA). Each timepoint has 3 biological replicates. All collected samples were frozen in liquid nitrogen as soon as they were separated from the plants and stored in a − 80 °C environment.

#### Library construction and RNA-sequencing

A total amount of 1 µg RNA per sample was used as input material for the RNA sample preparations. Sequencing libraries were generated using NEBNext® Ultra™ RNA Library Prep Kit for Illumina® (NEB, USA). mRNA was purified from total RNA using poly-T oligo-attached magnetic beads. Fragmentation was carried out in NEBNext First Strand Synthesis Reaction Buffer (5X). As long as short cDNA fragments were purified, they were extended with nucleotide adenines. After adenylation of 3′ ends of DNA fragments, adapters ligation, size selection and PCR amplification were performed for the prepare of sequencing. RNA-seq were carried out by Illumina comprehensive next-generation sequencing technique.

#### Transcriptome sequences profiling

Quality control of the raw data was carried out by FastQC using default parameters (version 0.11.5). After quality control of the raw data, clean reads were aligned to the reference genome (https://cottonfgd.org/about/download/assembly/genome.Ghir.HAU.fa.gz) by HISAT2 (version 2.1.0) [29]. The uniquely mapped reads were subjected to stringtie (version 1.3.6) to quantify the gene abundance [30]. FPKM value of all genes during fiber development was provided in our GitHub repository (https://github.com/LiuShang-777/SingleCNN/all_fpkm.txt.)

### Input data preprocessing

To check consistence of three biological replicates, we calculated Pearson coefficients among samples by pandas (version 0.23.4) and visualized Pearson coefficients in heatmap through seaborn (version 0.9.0). Classification of samples was performed by the t-SNE package in R language [31]. Input sequences were labeled according to transcription abundance and the threshold of FPKM value is set as 1. Gene was labeled as expressed status if it’s FPKM larger than 1, while, it was labeled as low expressed status. In the same stage, genes having conflict transcription status among different timepoints were trimmed. 500 nts flank the TSS were extracted as input sequences with length of 1000. These input sequences were one-hot encoded, including 4 nucleotides (A, T, C, G) and N. All input sequences we used in this study were stored in our GitHub repository (https://github.com/LiuShang-777/SingleCNN/) and the file names were init_utr5.zip, elong_utr5.zip, and scw_utr5.zip, respectively.

### Model building and training

Convolutional neural networks which contained one convolution layer and one fully connected layer were built by TensorFlow (version 2.0.0). The structure of models consists of 1 convolutional layer, 1 max-pooling layer, 1 dropout layer, and 2 fully connected layers. The last fully connected layer is to make a softmax classification. The loss function in CNN models is binary cross-entropy. More specific parameters about model structure were recorded in our Github repository (https://github.com/LiuShang-777/single_cnn/blob/main/01para_test.sh).

We took a fivefold cross-validation strategy to train our convolutional neural network. 20% of input genes were selected as test data, and 4000 of the rest 80% genes were selected as validation set, while, the others were used to train the convolutional neural network. Accuracy and the area under the receiver operating characteristic curve were calculated to assess model performance. (https://github.com/LiuShang-777/single_cnn/blob/main/02model_evaluate.py).

### Model interpretation

All true positive sequences were selected for model interpretation. Normal interpretation was implemented by DeepLIFT (https://github.com/kundajelab/deeplift) [15]. For N-adjusted kernel transformation, we proposed several symbols to denote it. The original kernel parameter is a matrix with a 5 × L shape, and L represents for length of kernel. We denoted Oij as the value in the original kernel matrix. For i and j, i ∈ {0,1,2,3,4} corresponding five nucleotides A, T, C, G, N, and j ∈ {0, 1, 2, …, L}, represent for L length kernels in CNN. we regularized original parameters by the effects of cryptic nucleotides. Rij is regularized probability in final transformed PWM, Rij = $\frac{\mathrm{max}\left(0,\mathrm{ Oij}-\mathrm{O}4\mathrm{j}\right)}{\sum_{i=0}^{i=4}\mathrm{max}\left(0,\mathrm{ Oij}-\mathrm{O}4\mathrm{j}\right)}$, i ∈ {0, 1, 2, 3}, j ∈ {0, 1, 2, …, L}. If 4 types of nucleotides were all transformed into 0, then 0.25 will be set for 4 nucleotides indicating no nucleotide preference in such position. Regularized kernels could be recorded as PWM file and transformed into MEME-recognized motif file by the chen2meme package in MEME [21]. Scripts about model interpretation was stored at https://github.com/LiuShang-777/single_cnn/blob/main/04motif_trans.sh

### Construction of support vector machine

Support vector machine (SVM) model was built on sklearn package (version 0.19.2), the kernel function we used is rbf. (https://github.com/LiuShang-777/single_cnn/blob/main/SVM.py). Features of SVM model are motifs, and the number of motifs possessed by sequences was calculated from fimo results (threshold of q value was set to 0.1). Features detected from CNNs and MEME were used to re-predict expression status by SVM. Evaluation of SVM model consisted of four aspects, accuracy, precise rate, recall rate, and f1 score.

### Motif alignment and distribution scanning

Transformed kernels could be aligned to the JASPAR database through tomtom in MEME (v5.1.1). The motif database was downloaded from MEME (https://meme-suite.org/meme/meme-software/Databases/motifs/motif_databases.12.21.tgz). We set 0.05 as the q-value threshold in tomtom for detection of known motifs. However, q-value was set to 0.1 in tomtom for generation of non-redundant novel motifs. Aligned motifs were scanned along sequences without the reverse complementary strand by the fimo in MEME [21]. Scripts to perform motif alignment were stored at https://github.com/LiuShang-777/single_cnn/blob/main/05motif_align.sh and https://github.com/LiuShang-777/single_cnn/blob/main/07motif_unalign.sh.

### GO enrichment analysis

GO enrichment analyses were performed by R package clusterprofile [32]. GO database was constructed by eggNOG-mapper (http://eggnog5.embl.de/). 30 enriched GO terms in with minimum q value were selected for visualization.

## Supplementary Information

**Additional file 1.** Title of data: Heatmap of correlationships between all 33 samples. Description of data: Numerics presented on heatmap were coefficients between each two samples. Deeper red color represents stronger correlationship.

**Additional file 2.** Title of data: t-SNE analysis on 11 time points. Description of data: Mean FPKM of three replicates was calculated for t-SNE analysis. All 11 time points were classified into three clusters. Three colors represent for three fiber development stages.

**Additional file 3.** Title of data: Accuracies of models with different parameters in initiation. Description of data: We tested performance of models with various parameters (filter number, kernel size and max-pooling size). Accuracies of these models were calculated to select the best model.

**Additional file 4.** Title of data: Accuracies of models with different parameters in elongation. Description of data: We tested performance of models with various parameters (filter number, kernel size and max-pooling size). Accuracies of these models were calculated to select the best model.

**Additional file 5.** Title of data: Accuracies of models with different parameters in SCW. Description of data: We tested performance of models with various parameters (filter number, kernel size and max-pooling size). Accuracies of these models were calculated to select the best model.

**Additional file 6.** Title of data: Effects visualization for models built in elongation. Description of data: Effect of each loc within input sequences were calculated by DeepLift. Five colors represent for four kinds of nucleotides and cryptic nucleotides in genome. 10 nts from upstream and downstream were displayed seperately in dotplot.

**Additional file 7.** Title of data: Effects visualization for models built in SCW. Description of data: Effect of each loc within input sequences were calculated by DeepLIFT. Five colors represent for four kinds of nucleotides and cryptic nucleotides in genome. 10 nts from upstream and downstream were displayed seperately in dotplot.

**Additional file 8.** Title of data: Output value of kernels in model 0 during fiber initiation. Description of data: Red pots for expressed genes while blue pots for low expressed genes.

**Additional file 9.** Title of data: Output value of kernels in model 1 during fiber initiation. Description of data: Red pots for expressed genes while blue pots for low expressed genes.

**Additional file 10.** Title of data: Output value of kernels in model 2 during fiber initiation. Description of data: Red pots for expressed genes while blue pots for low expressed genes.

**Additional file 11.** Title of data: Output value of kernels in model 3 during fiber initiation. Description of data: Red pots for expressed genes while blue pots for low expressed genes.

**Additional file 12.** Title of data: Output value of kernels in model 4 during fiber initiation. Description of data: Red pots for expressed genes while blue pots for low expressed genes.

**Additional file 13.** Title of data: Output value of kernels in model 0 during fiber elongation. Description of data: Red pots for expressed genes while blue pots for low expressed genes.

**Additional file 14.** Title of data: Output value of kernels in model 1 during fiber elongation. Description of data: Red pots for expressed genes while blue pots for low expressed genes.

**Additional file 15.** Title of data: Output value of kernels in model 2 during fiber elongation. Description of data: Red pots for expressed genes while blue pots for low expressed genes.

**Additional file 16.** Title of data: Output value of kernels in model 3 during fiber elongation. Description of data: Red pots for expressed genes while blue pots for low expressed genes.

**Additional file 17.** Title of data: Output value of kernels in model 4 during fiber elongation. Description of data: Red pots for expressed genes while blue pots for low expressed genes.

**Additional file 18.** Title of data: Output value of kernels in model 0 during SCW. Description of data: Red pots for expressed genes while blue pots for low expressed genes.

**Additional file 19.** Title of data: Output value of kernels in model 1 during SCW. Description of data: Red pots for expressed genes while blue pots for low expressed genes.

**Additional file 20.** Title of data: Output value of kernels in model 2 during SCW. Description of data: Red pots for expressed genes while blue pots for low expressed genes.

**Additional file 21.** Title of data: Output value of kernels in model 3 during SCW. Description of data: Red pots for expressed genes while blue pots for low expressed genes.

**Additional file 22.** Title of data: Output value of kernels in model 4 during SCW. Description of data: Red pots for expressed genes while blue pots for low expressed genes.

**Additional file 23.** Title of data: Heatmap for effects of N in models during fiber initiation. Description of data: Values annotated in the heatmap indicate the order of N sorted by effects of 5 characters in sequences. 4 means N has the largest effect and 0 means effects of N is minimum among 5 characters. Effects of N in five models were visualized.

**Additional file 24.** Title of data: Heatmap for effects of N in models during fiber elongation. Description of data: Values annotated in the heatmap indicate the order of N sorted by effects of 5 characters in sequences. 4 means N has the largest effect and 0 means effects of N is minimum among 5 characters. Effects of N in five models were visualized.

**Additional file 25.** Title of data: Heatmap for effects of N in models during SCW. Description of data: Values annotated in the heatmap indicate the order of N sorted by effects of 5 characters in sequences. 4 means N has the largest effect and 0 means effects of N is minimum among 5 characters. Effects of N in five models were visualized.

**Additional file 26.** Title of data: Venn visualization for detected known motifs. Description of data: Motifs appeared in more than 2 models (among 5 cross validation models) were extracted in 3 stages. 10 motifs were shared by three stages.

**Additional file 27.** Title of data: Motifs detected by CNN interpretation and MEME. Description of data: Motifs-binding proteins interpreted from CNNs were listed and ID of motifs in MEME database were also listed, with corresponding number of those detected from CNN in each stage.

**Additional file 28.** Title of data: protein model templates of 6 DOF genes. Description of data: Protein models of 6 DOFs were built by SwissModel, and the best 50 templates were selected and listed in this table.

**Additional file 29.** Title of data: Distribution of a novel sequence feature from initiation models. Description of data: Red for expressed genes and blue for low expressed genes.

**Additional file 30.** Title of data: Distribution of a novel sequence feature from elongation models. Description of data: Red for expressed genes and blue for low expressed genes.

**Additional file 31.** Title of data: Distribution of a novel sequence feature from SCW models. Description of data: Red for expressed genes and blue for low expressed genes.

**Additional file 32.** Title of data: GO enrichment on genes possessed novel motif detected during fiber initiation. Description of data: First ten the most enriched GO terms in biological process, molecular function, cell component, respectively were selected for visualization.

**Additional file 33.** Title of data: GO enrichment on genes possessed novel motif detected during fiber elongation. Description of data: First ten the most enriched GO terms in biological process, molecular function, cell component, respectively were selected for visualization.

**Additional file 34.** Title of data: GO enrichment on genes possessed novel motif detected during SCW. Description of data: First ten the most enriched GO terms in biological process, molecular function, cell component, respectively were selected for visualization.

## Data Availability

Genome sequences (*G. hirsutum.* L, HAU) we used in this study is downloaded from CottonFGD (https://cottonfgd.org/about/download.html). Transcriptional abundance of all genes were available at github repository (https://github.com/LiuShang-777/SingleCNN/all_fpkm.txt). Source for analysis in this study could gained from https://github.com/LiuShang-777/single_cnn/. RNA-seq data used in this study is still unpublished, researchers could contact with corresponding authors (zdy041@163.com) if raw data is in demand.

## Abbreviations

SCW: Secondary cell wall thickening
CNN: Convolutional neural network
TSS: Transcription start site
UTR: Untranslated region
AUROC: Area under the receiver operating characteristic curve
PWM: Position weight matrix
DPA: Day post-anthesis
nt: Nucleotides
FPKM: Fragments Per Kilobase of exon model per Million mapped fragments
CHIP-seq: Chromatin immunoprecipitation sequencing
CLIP: UV-crosslinked immunoprecipitation
GO: Gene ontology
SVM: Support vector machine
