# Supplementary figures and images for: Interpretation of convolutional neural networks reveals crucial sequence features involving in transcription during fiber development
Source: BMC Bioinformatics. 2022 Mar 15;23:91. doi: 10.1186/s12859-022-04619-9 (PMC8922751; doi:10.1186/s12859-022-04619-9)

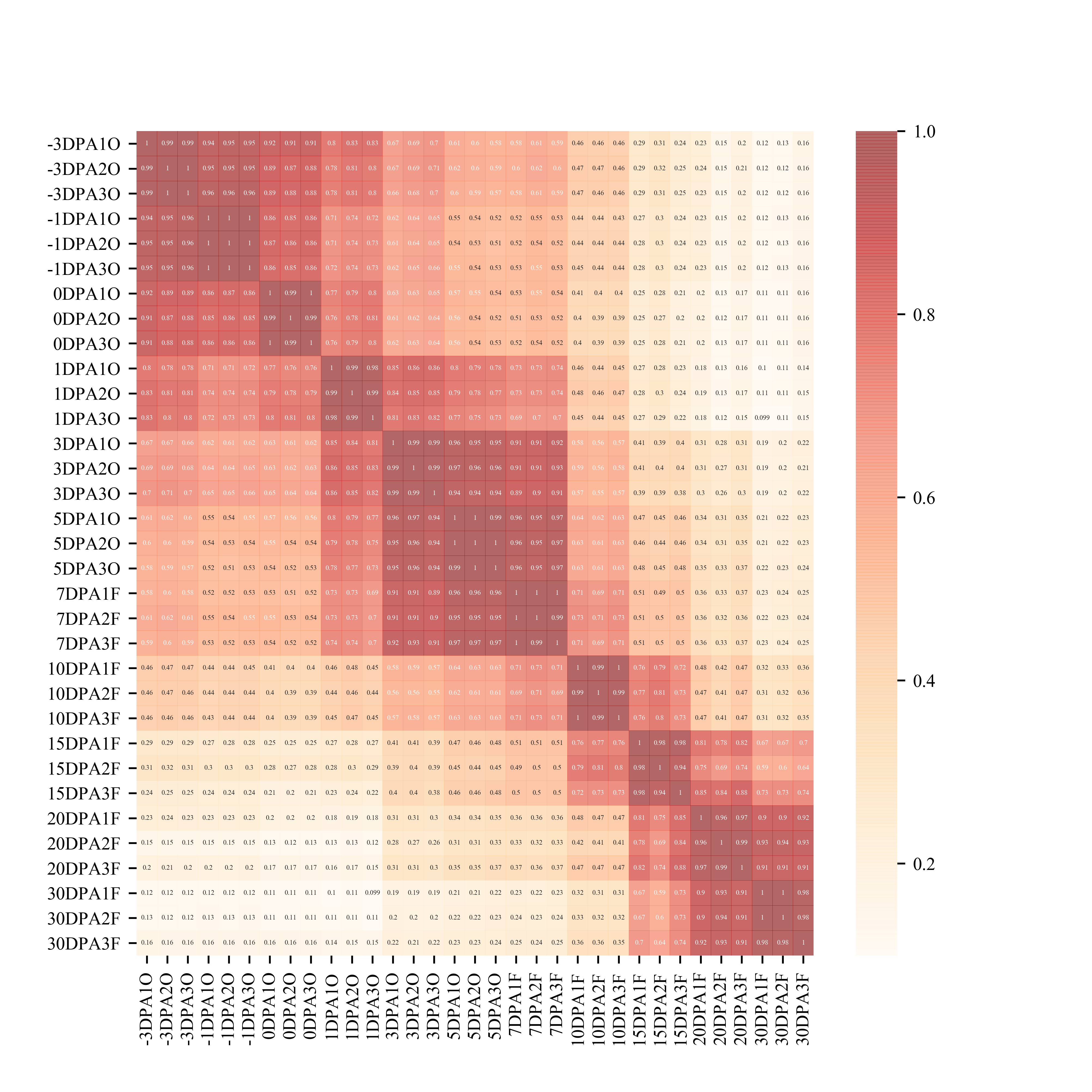

Supplement: Supplementary file 1 — Additional file 1. Title of data: Heatmap of correlationships between all 33 samples. Description of data: Numerics presented on heatmap were coefficients between each two samples. Deeper red color represents stronger correlationship. [file 12859_2022_4619_MOESM1_ESM.png]

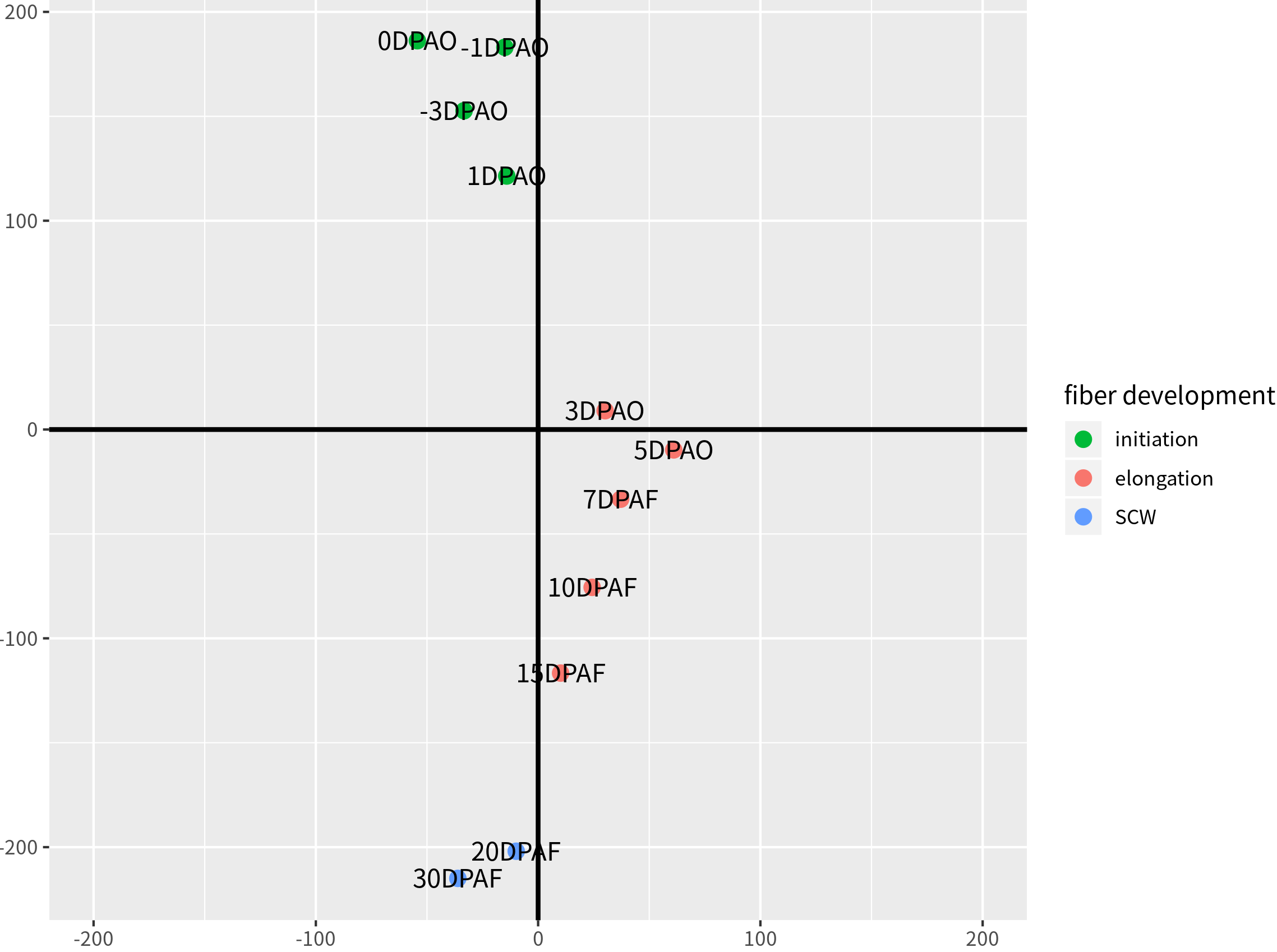

Supplement: Supplementary file 2 — Additional file 2. Title of data: t-SNE analysis on 11 time points. Description of data: Mean FPKM of three replicates was calculated for t-SNE analysis. All 11 time points were classified into three clusters. Three colors represent for three fiber development stages. [file 12859_2022_4619_MOESM2_ESM.png]

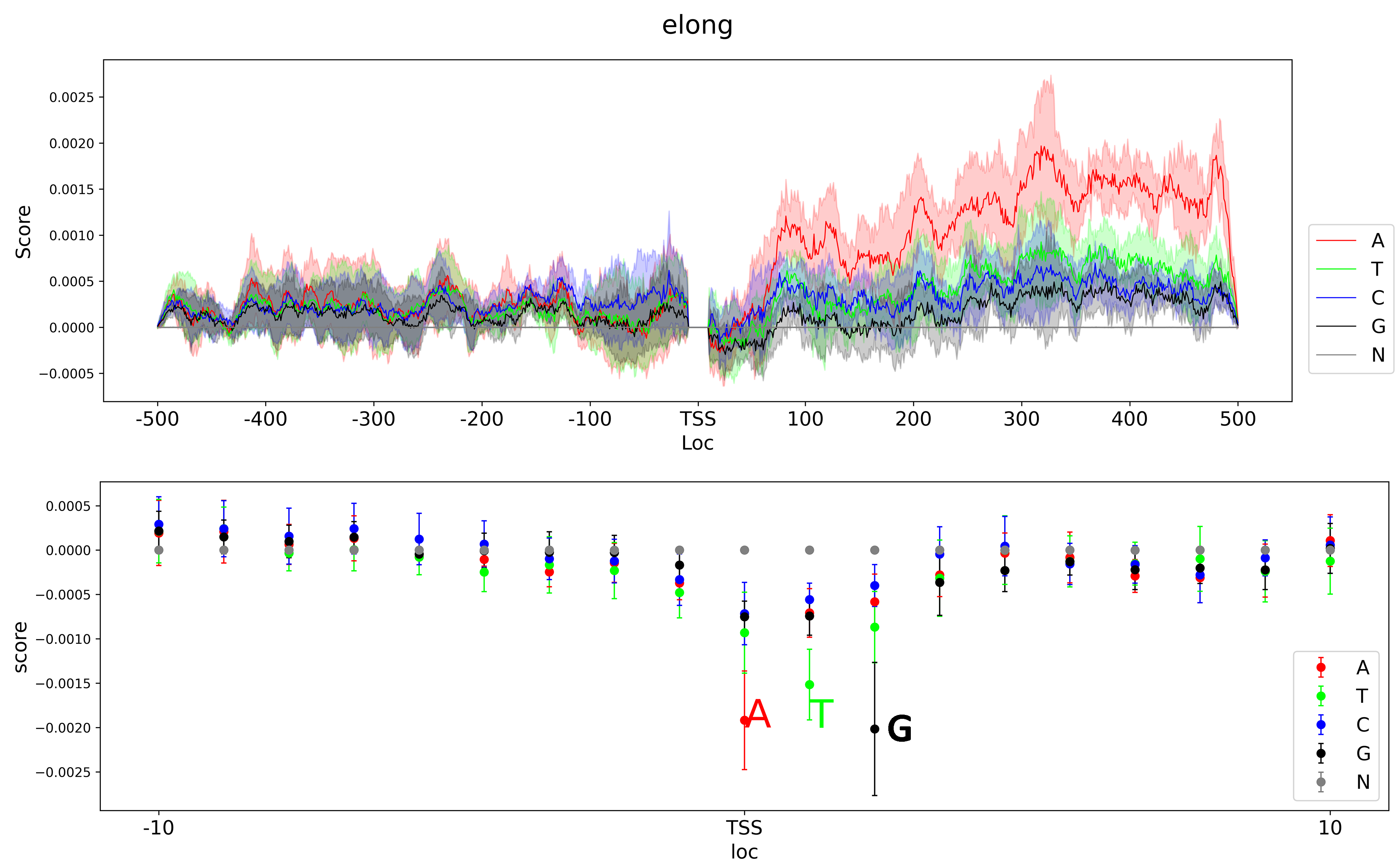

Supplement: Supplementary file 6 — Additional file 6. Title of data: Effects visualization for models built in elongation. Description of data: Effect of each loc within input sequences were calculated by DeepLift. Five colors represent for four kinds of nucleotides and cryptic nucleotides in genome. 10 nts from upstream and downstream were displayed seperately in dotplot. [file 12859_2022_4619_MOESM6_ESM.png]

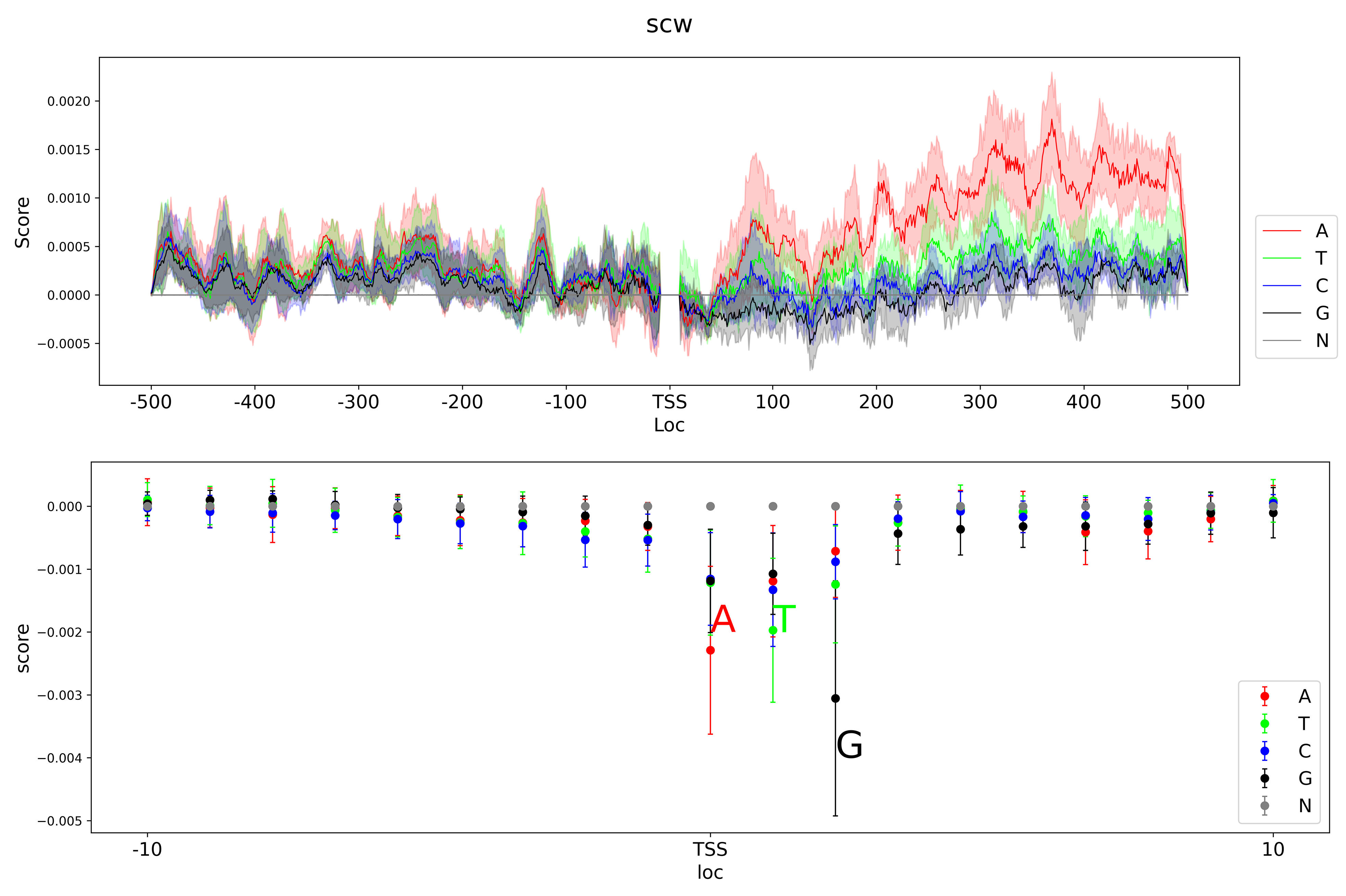

Supplement: Supplementary file 7 — Additional file 7. Title of data: Effects visualization for models built in SCW. Description of data: Effect of each loc within input sequences were calculated by DeepLIFT. Five colors represent for four kinds of nucleotides and cryptic nucleotides in genome. 10 nts from upstream and downstream were displayed seperately in dotplot. [file 12859_2022_4619_MOESM7_ESM.png]

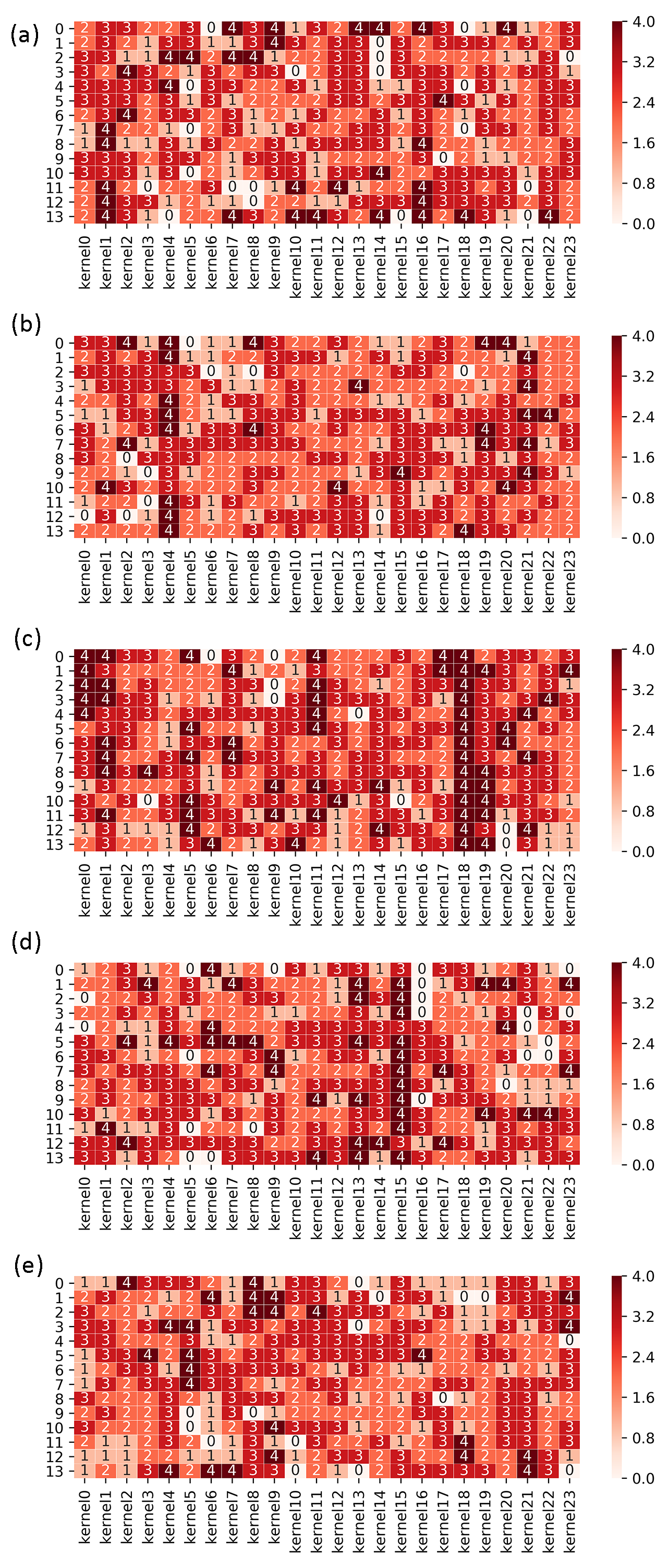

Supplement: Supplementary file 23 — Additional file 23. Title of data: Heatmap for effects of N in models during fiber initiation. Description of data: Values annotated in the heatmap indicate the order of N sorted by effects of 5 characters in sequences. 4 means N has the largest effect and 0 means effects of N is minimum among 5 characters. Effects of N in five models were visualized. [file 12859_2022_4619_MOESM23_ESM.png]

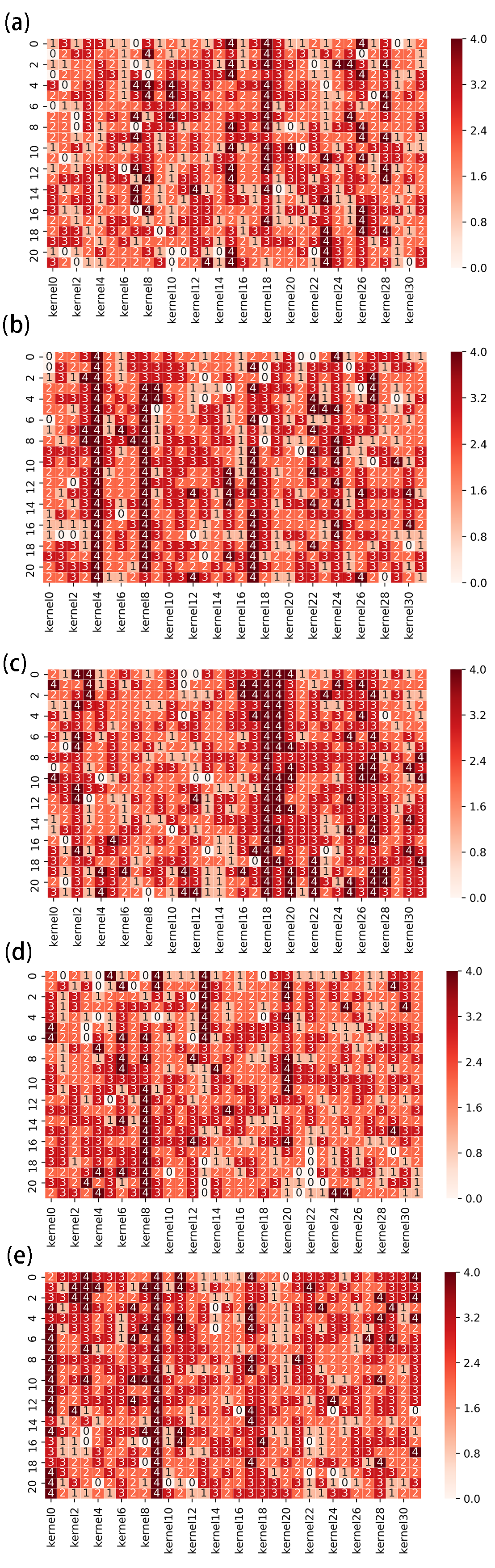

Supplement: Supplementary file 24 — Additional file 24. Title of data: Heatmap for effects of N in models during fiber elongation. Description of data: Values annotated in the heatmap indicate the order of N sorted by effects of 5 characters in sequences. 4 means N has the largest effect and 0 means effects of N is minimum among 5 characters. Effects of N in five models were visualized. [file 12859_2022_4619_MOESM24_ESM.png]

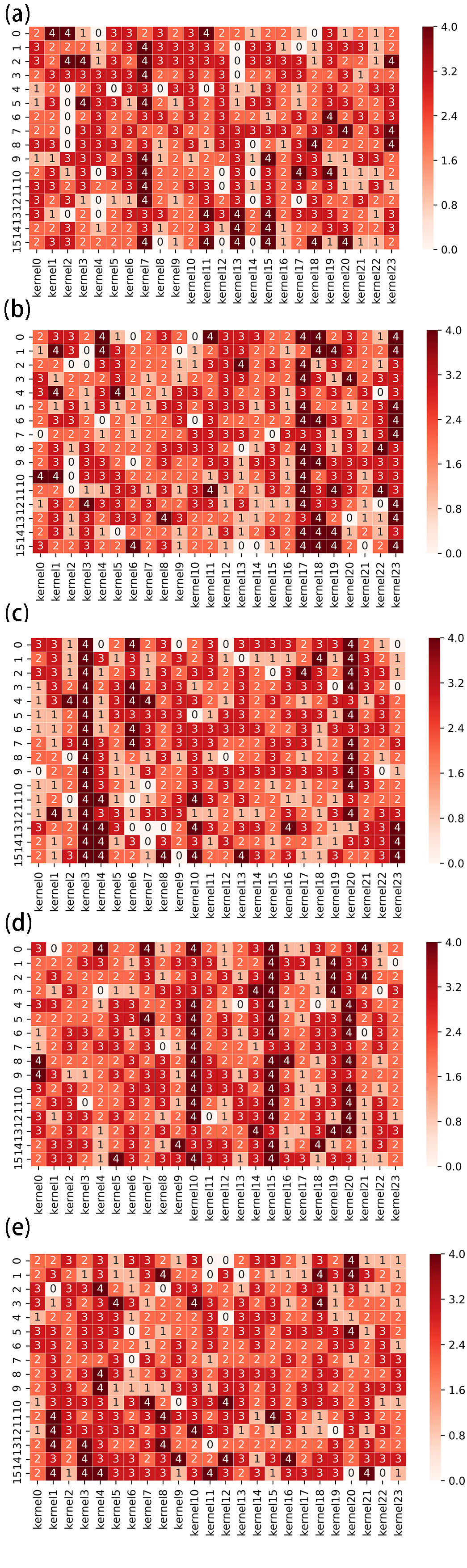

Supplement: Supplementary file 25 — Additional file 25. Title of data: Heatmap for effects of N in models during SCW. Description of data: Values annotated in the heatmap indicate the order of N sorted by effects of 5 characters in sequences. 4 means N has the largest effect and 0 means effects of N is minimum among 5 characters. Effects of N in five models were visualized. [file 12859_2022_4619_MOESM25_ESM.png]

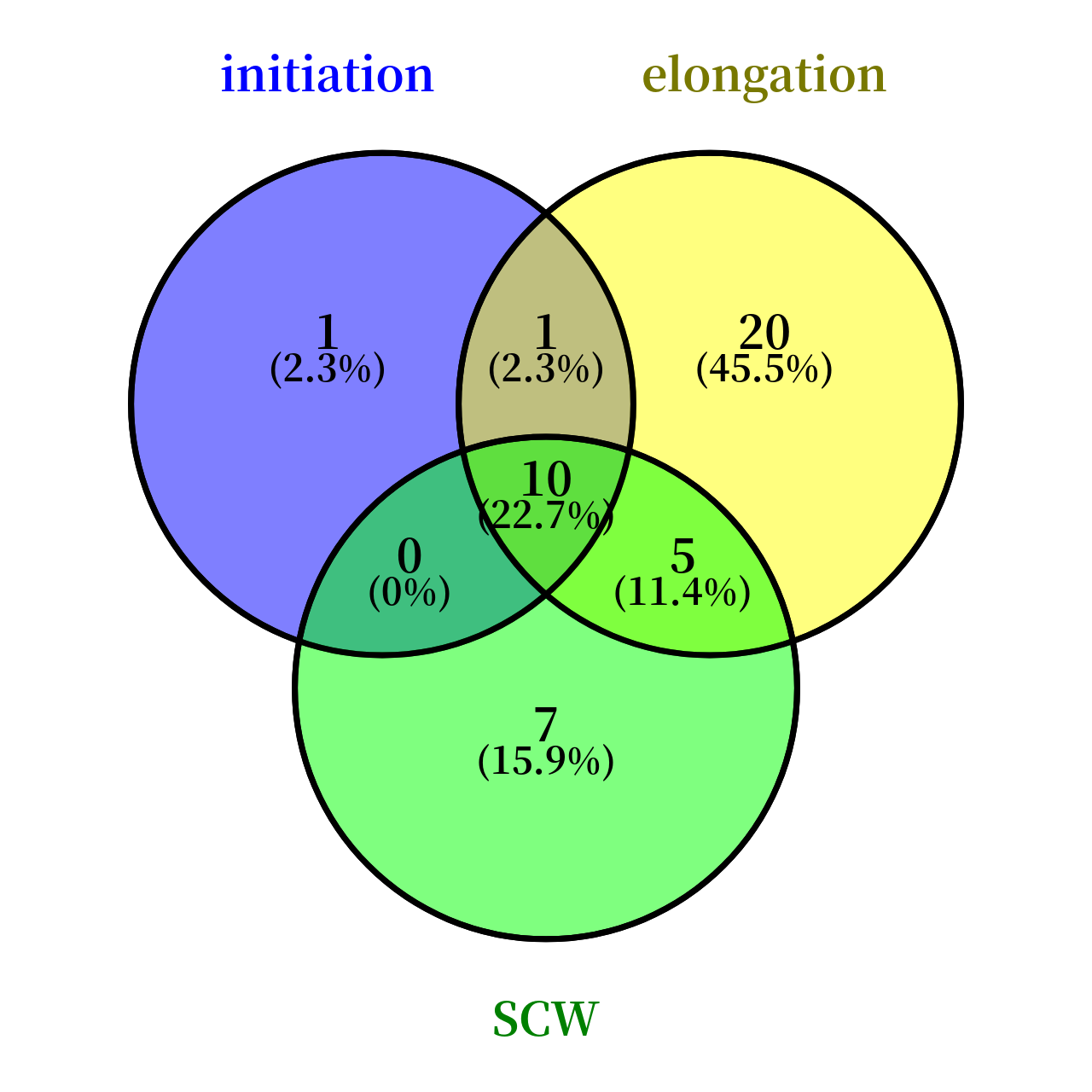

Supplement: Supplementary file 26 — Additional file 26. Title of data: Venn visualization for detected known motifs. Description of data: Motifs appeared in more than 2 models (among 5 cross validation models) were extracted in 3 stages. 10 motifs were shared by three stages. [file 12859_2022_4619_MOESM26_ESM.png]

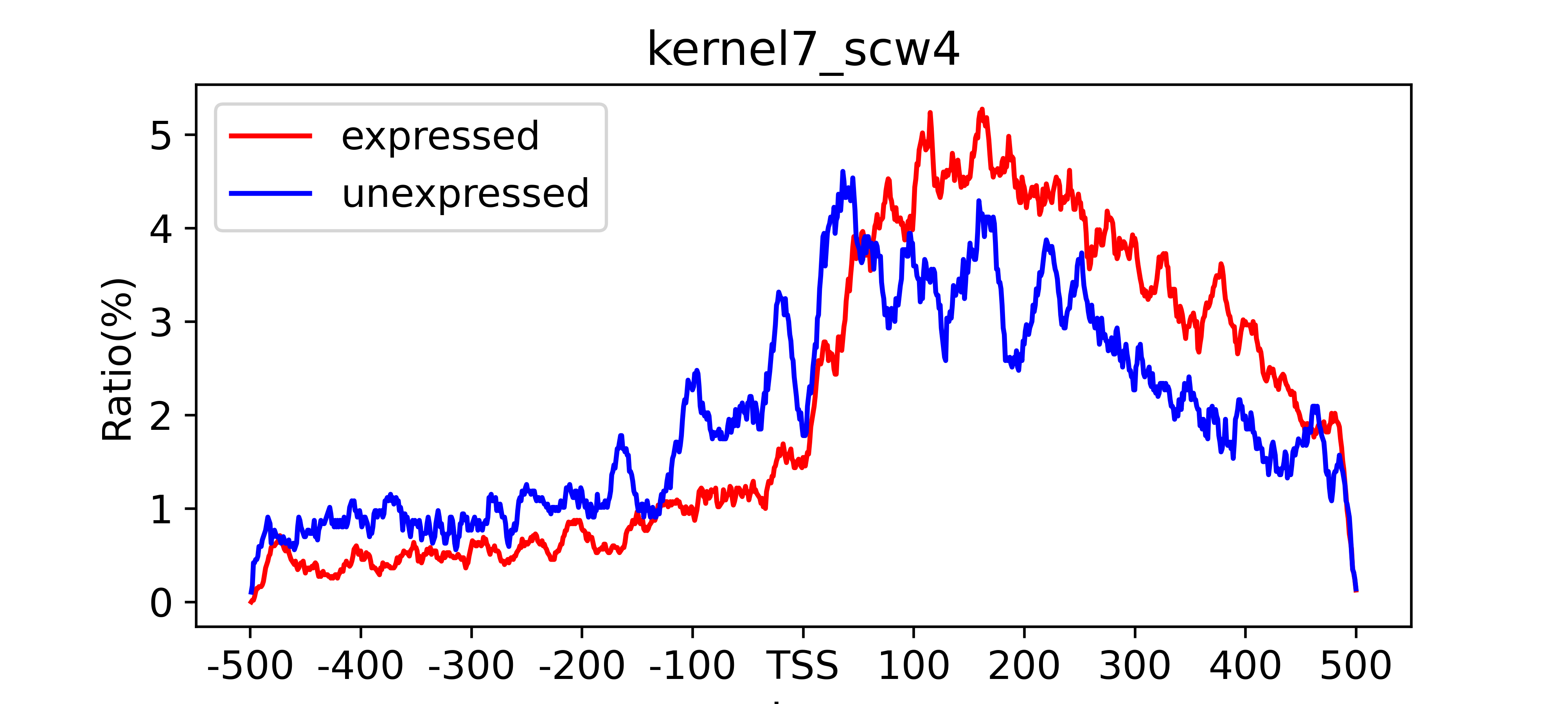

Supplement: Supplementary file 29 — Additional file 29. Title of data: Distribution of a novel sequence feature from initiation models. Description of data: Red for expressed genes and blue for low expressed genes. [file 12859_2022_4619_MOESM29_ESM.png]

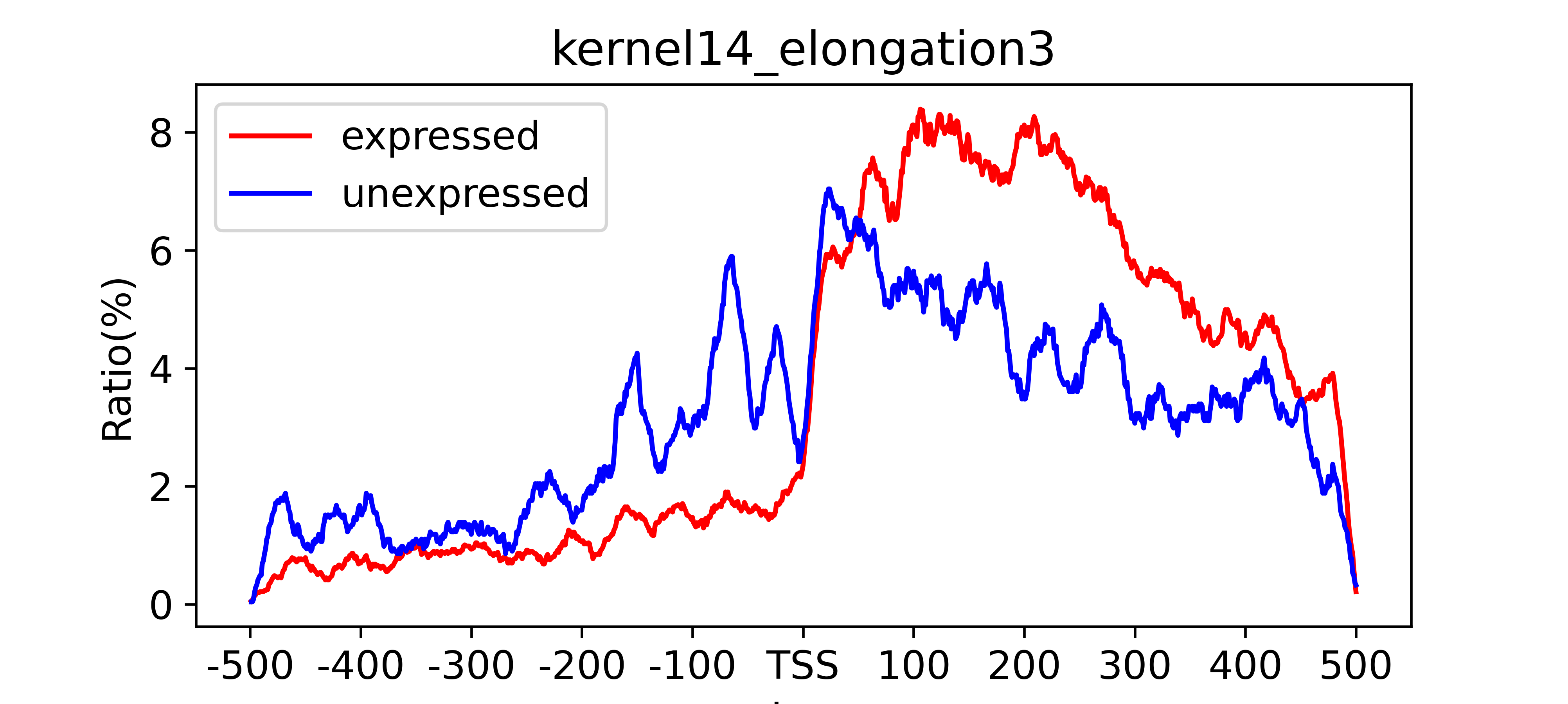

Supplement: Supplementary file 30 — Additional file 30. Title of data: Distribution of a novel sequence feature from elongation models. Description of data: Red for expressed genes and blue for low expressed genes. [file 12859_2022_4619_MOESM30_ESM.png]

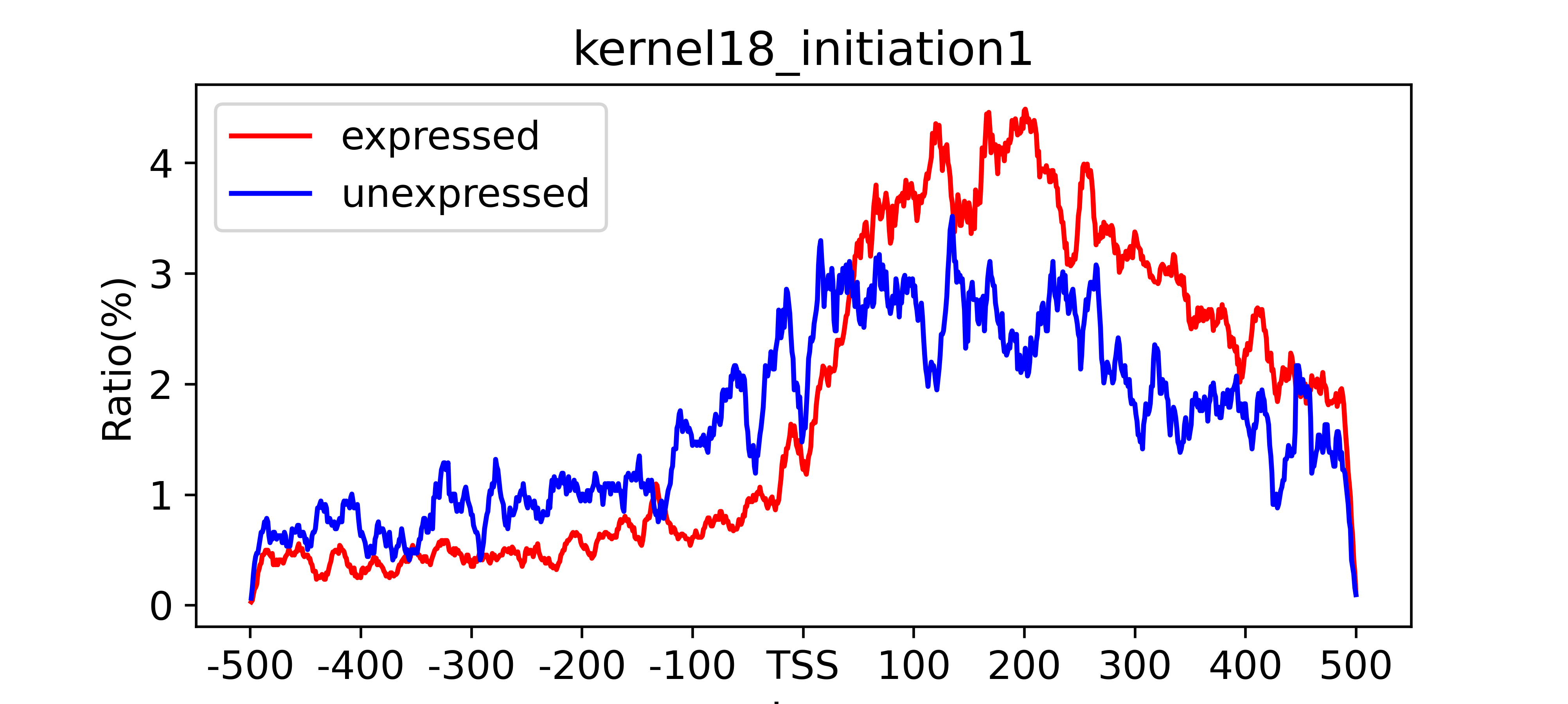

Supplement: Supplementary file 31 — Additional file 31. Title of data: Distribution of a novel sequence feature from SCW models. Description of data: Red for expressed genes and blue for low expressed genes. [file 12859_2022_4619_MOESM31_ESM.png]

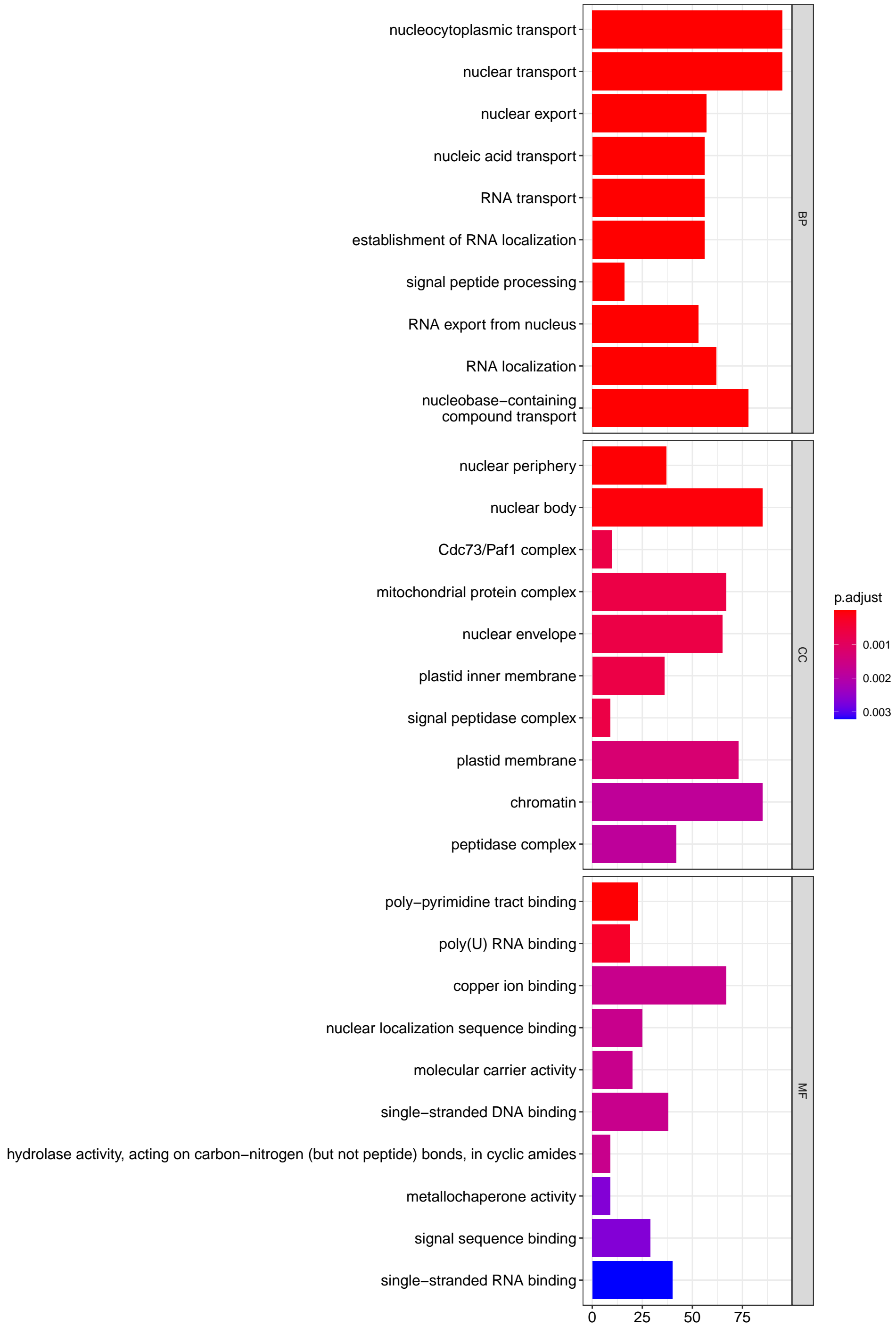

Supplement: Supplementary file 32 — Additional file 32. Title of data: GO enrichment on genes possessed novel motif detected during fiber initiation. Description of data: First ten the most enriched GO terms in biological process, molecular function, cell component, respectively were selected for visualization. [file 12859_2022_4619_MOESM32_ESM.pdf]

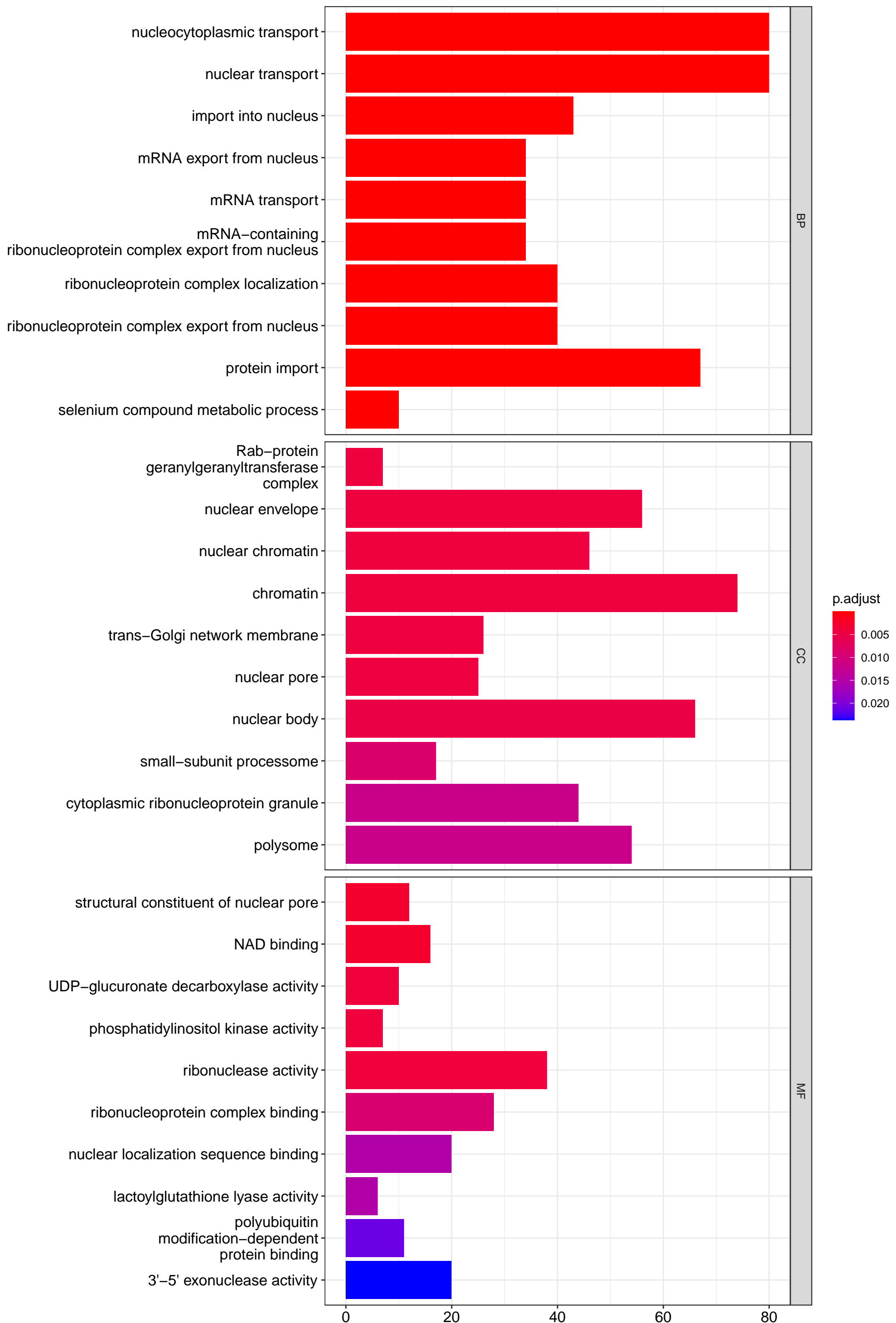

Supplement: Supplementary file 33 — Additional file 33. Title of data: GO enrichment on genes possessed novel motif detected during fiber elongation. Description of data: First ten the most enriched GO terms in biological process, molecular function, cell component, respectively were selected for visualization. [file 12859_2022_4619_MOESM33_ESM.pdf]

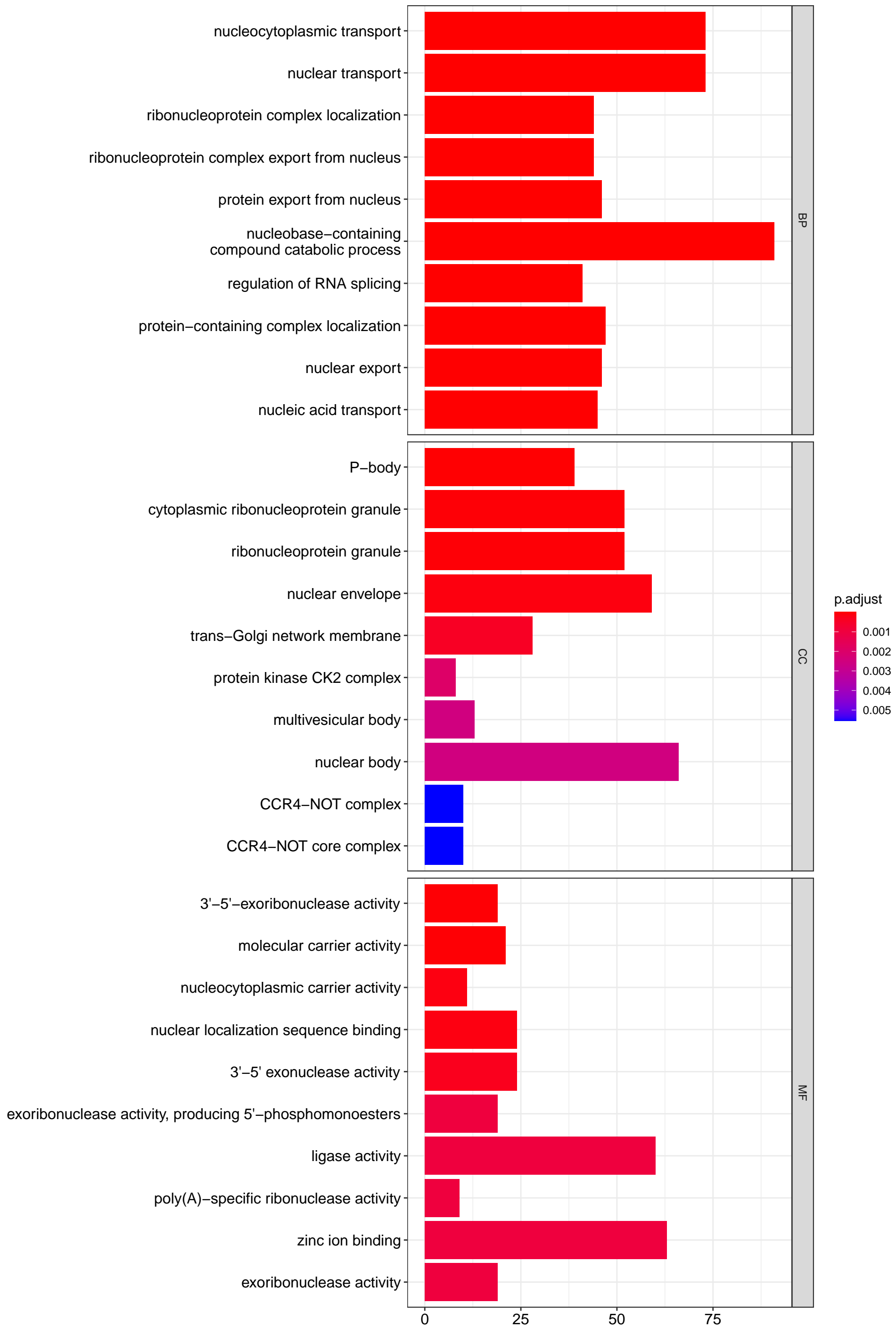

Supplement: Supplementary file 34 — Additional file 34. Title of data: GO enrichment on genes possessed novel motif detected during SCW. Description of data: First ten the most enriched GO terms in biological process, molecular function, cell component, respectively were selected for visualization. [file 12859_2022_4619_MOESM34_ESM.pdf]
